# Statistical Characterization of Food-Derived α-Amylase Inhibitory Peptides: Computer Simulation and Partial Least Squares Regression Analysis

**DOI:** 10.3390/molecules29020395

**Published:** 2024-01-13

**Authors:** Wenhui Li, Shangci Yang, Jiulong An, Min Wang, He Li, Xinqi Liu

**Affiliations:** 1 China Food Flavor and Nutrition Health Innovation Center, Beijing Technology and Business University (BTBU), Beijing 100048, China; 2050201012@st.btbu.edu.cn (W.L.); yangshangci@126.com (S.Y.); anjiulong0322@163.com (J.A.); balance0831@163.com (M.W.); liuxinqi@btbu.edu.cn (X.L.); 2 University of Chinese Academy of Sciences, Beijing 100049, China; 3 National Soybean Processing Industry Technology Innovation Center, Beijing Technology and Business University (BTBU), Beijing 100048, China

**Keywords:** α-amylase, α-amylase inhibitory peptide, molecular docking, molecular dynamics, partial least squares regression, hydrogen bonds

## Abstract

α-Amylase inhibitory peptides are used to treat diabetes, but few studies have statistically characterized their interaction with α-amylase. This study performed the molecular docking of α-amylase with inhibitory peptides from published papers. The key sites, side chain chargeability, and hydrogen bond distribution characteristics were analyzed. Molecular dynamics simulated the role of key sites in complex stability. Moreover, partial least squares regression (PLSR) was used to analyze the contribution of different amino acids in the peptides to inhibition. The results showed that, for the α-amylase molecule, His201 and Gln63, with the highest interaction numbers (INs, 15, 15) and hydrogen bond values (HBVs, 11.50, 10.33), are the key sites on α-amylase, and amino acids with positively charged side chains were important for inhibitory activity. For the inhibitory peptides, Asp and Arg had the highest HBVs, and amino acids with charged side chains were more likely to form hydrogen bonds and exert inhibitory activity. In molecular dynamics simulations, peptides involving key binding sites formed more stable complexes with α-amylase than α-amylase alone, suggesting enhanced inhibitory effects. Further, PLSR results showed that amino acids close to the N-terminus of the inhibitory peptide, located in the third and fifth positions, were significantly correlated with its inhibitory activity. In conclusion, this study provides a new approach to developing and screening α-amylase inhibitors.

## 1. Introduction

Diabetes has become a significant threat to human health, with 536.6 million sufferers between the ages of 20 and 79, accounting for 10.5% of the population in 2021. This is expected to increase to 783.2 million by 2045, according to data published by the International Diabetes Federation [1]. α-Amylase is a key enzyme involved in the digestion and absorption of carbohydrates, hydrolyzing starch into maltose or glucose in the mouth and pancreas [2]. The inhibition of α-amylase activity can effectively control blood glucose and, thus, alleviate diabetes. Currently, several studies have isolated amylase inhibitory peptide fractions and hydrolysates from easy- and hard-to-cook beans (*Phaseolus vulgaris* L.) [3], *pinto* bean protein isolate [4], oat protein [5], *perilla* seed meal protein [6], black cricket protein [7], and *Nephelium lappacheum* and *mutabile* seeds [8], with some even identifying potential α-amylase inhibitory peptide sequences. Some peptides have been synthesized into pure products, revealing their α-amylase inhibitory activity in vitro and antidiabetic drug potential. Although an increasing number of α-amylase inhibitory peptides have been identified, little is known about their statistical commonalities, while the mechanisms behind α-amylase inhibition at the amino acid and physicochemical levels also remain unclear. The development of antidiabetic medications has been constrained by the inefficiency and lack of systematicity of these studies. Examining the statistical characteristics of existing α-amylase inhibitory peptides is necessary to optimize the development and design of peptide drugs for enzyme inhibition.

**Figure 1:**
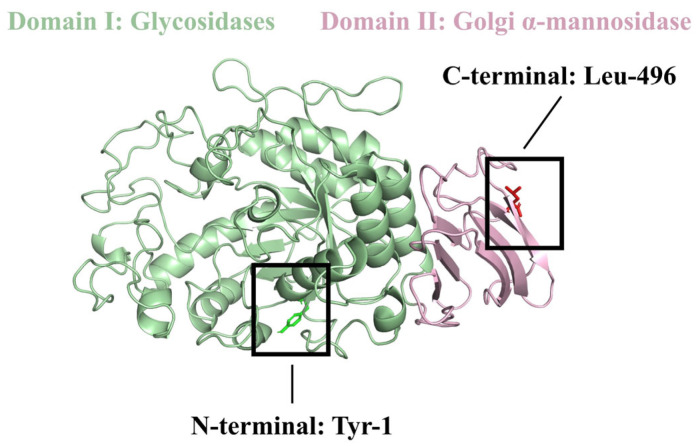
Schematic diagram of α-amylase.

Computer simulation techniques have been widely used in ligand screening and mechanistic studies [9]. Molecular docking simulates the interactions between proteins and ligands, deriving the most likely binding state based on intermolecular interaction forces [10]. Molecular dynamics simulates the behavior of biological systems over time to determine the equilibrium state of a complex [11]. Both techniques have been used to study the mechanisms of angiotensin-converting enzymes (ACEs) [12], pancreatic lipase [13], myeloperoxidase (MPO) [14], and α-glucosidase [15], suggesting that both techniques can be used to systematically explore the possibility of α-amylase-inhibiting peptide activity. For α-amylase, a schematic diagram (Figure 1) was obtained according to the RCSB protein database (https://www.rcsb.org/, accessed on 27 December 2023). α-Amylase has Tyr-1 at the N-terminus and Leu-496 at the C-terminus, and it mainly consists of two structural domains: the green area represents structural domain 1 Glycosidase, and the pink area represents structural domain 2 Golgi α-mannosidase. Molecular docking and molecular dynamics simulation of α-amylase and inhibitory peptides can better explore the key site and mechanism of the inhibitory peptide function.

Using statistical methods to analyze the results of molecular docking helps to find regularities in enzyme–peptide interactions. Additional studies on inhibitory peptide amino acid residues that are more likely to interact with α-amylase or establish hydrogen bonds are also necessary. Amino acids display different physicochemical characteristics, including hydrophobic, electrical, steric, and hydrogen bond contributory properties. Partial least squares regression (PLSR), one of the most efficient mathematical methods for analyzing correlations between multiple variables and a dependent variable, can be used to investigate the influence of multiple factors on the dependent variable and quantitatively study the association between these physicochemical peptide properties and their inhibitory effects.

This study aimed to investigate the statistical characterization of different food-derived α-amylase inhibitory peptides. Molecular docking was performed to simulate α-amylase with twenty inhibitory peptides, followed by the analysis of key sites and amino acid side-chain-charged properties of α-amylase and peptides, respectively. Three peptides containing the key sites were then selected for further molecular dynamics to verify the role of the key sites, and finally, PLSR was performed to further analyze the contribution of different amino acids on the peptides to their inhibitory activity. This study helps to address the knowledge gap in the general mechanism of action of α-amylase inhibitory peptides and provides theoretical guidance for the development of functional foods and antidiabetic drugs.

## 2. Results and Discussion

### 2.1. Molecular Docking Result Analysis of α-Amylase Molecule and Its Inhibitory Peptides

**Figure 2:**
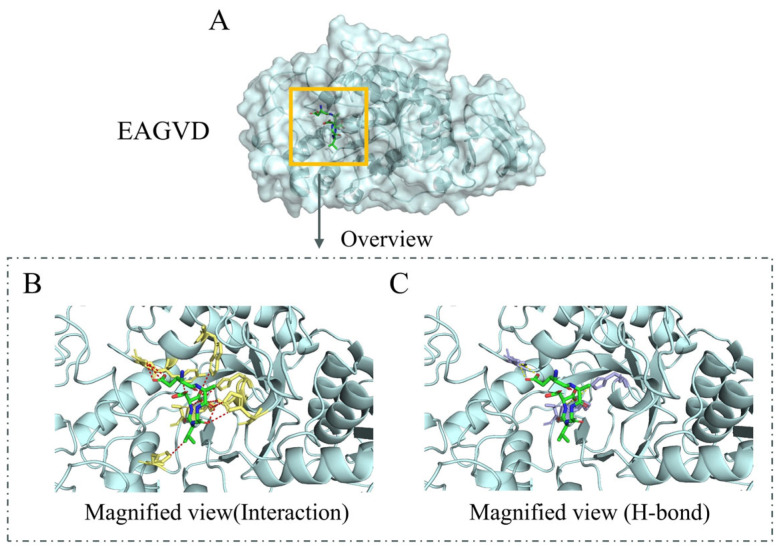
Molecular docking of EAGVD with α-amylase visualization images. (**A**) Overview. Blue molecules represent α-amylase; green molecules are peptide fragments. (**B**) Magnified view (interaction). Yellow molecules represent amino acids on α-amylase that interact with peptide segments; red dashed lines indicate interactions. (**C**) Magnified view (hydrogen bond). Purple molecules represent amino acids on α-amylase that form hydrogen bonds with the peptide segment, and yellow dashed lines indicate interactions.

Twenty α-amylase inhibitory peptides were screened from a large number of studies (Table 1), all of which were food-derived and significantly inhibited α-amylase activity. Taking the EAGVD peptide as an example, molecular docking was simulated with α-amylase, and the docking results are shown in Table S1. There are nine conformations in total, which are ranked according to affinity. Negative affinities indicate spontaneous binding; larger absolute values mean stronger binding (which can be considered a binding score); and the conformation with the highest binding score was finally selected. Figure 2A,B demonstrate the molecular docking poses of the EAGVD peptide with α-amylase, and how EAGVD interacts with Trp58, Tyr62, Gln63, Arg195, Asp197, His201, His299, Asp300, and His305 on α-amylase (Table S2). The formation of hydrogen bonds during the combination of the two was also analyzed. Figure 2C shows the hydrogen bonds generated by the combination of EAGVD and α-amylase, with a total of five, and the specific locations and hydrogen bond lengths are shown in Table S3. The molecular docking results for the remaining 19 peptides with α-amylase are also presented in Figure S1 and Tables S1–S3.

### 2.2. Analysis of the Key Sites on the α-Amylase Molecules

#### 2.2.1. Analysis of the Docking Site Interaction Frequency on the α-Amylase Molecule

By counting the frequency of docking site actions on α-amylase, the key site of inhibitory peptide action can be identified better. As shown in Table 2, 69 amino acid residues on the α-amylase molecule were involved in docking. The largest interaction numbers (INs) were His201, Gln63, Asp300, and His305, with values of 15, 15, 14, and 14, respectively. The results showed that the inhibitory peptides were more likely to interact with His201, Gln63, Asp300, and His305 on α-amylase.

#### 2.2.2. Analysis of the Docking Site Hydrogen Bond Qualities of α-Amylase Molecules

Studies have shown that hydrogen bond formation is vital for the inhibitory effect of acarbose, flavonoids [22], and plant protein inhibitors [23] on α-amylase. Multiple hydrogen bonds may play an important role in the interaction between peptide ligands and target proteins in the rational design of affinity peptides [24]. Other studies involving the α-amylase inhibitory activity of bitter peptides in yak cheese have shown that hydrogen bonds are stable during enzyme–peptide binding and that their number and bond lengths can determine peptide affinity [25]. Therefore, we suggest that the formation of high-quality hydrogen bonds (more hydrogen bonds, shorter bond lengths) during the binding of the inhibitory peptide to α-amylase will contribute to the binding of the two.

Consequently, a variable that fully accounts for the number and length of hydrogen bonds was designed to describe the hydrogen bond mass, namely, the hydrogen bond value (HBV). As shown in Table 3, Gln63, His201, and Arg252 represented the amino acid residues with the highest HBVs on the α-amylase molecule. The results indicated that the inhibitory peptides were more likely to form hydrogen bonds with Gln63, His201, and Arg252 on α-amylase and exert inhibitory activity.

#### 2.2.3. Combined IN and HBV Analysis of the Amino Acid Sites of the α-Amylase Molecule

**Figure 3:**
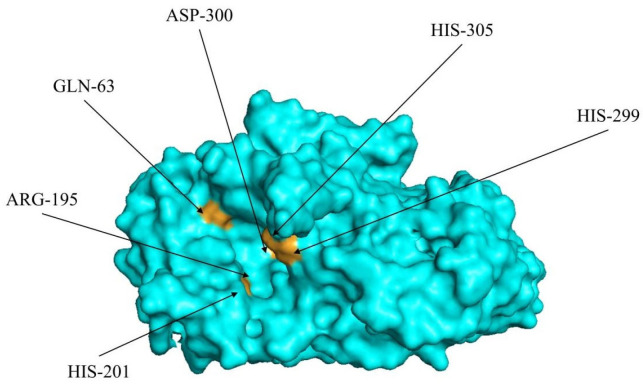
Schematic representation of the positions of the six important amino acids on the α-amylase molecule that bind to the inhibitory peptide (based on IN and HBV results).

The IN and HBV results were combined for further analysis. From an amino acid residue perspective, His201 and Gln63 were the most important docking sites in terms of both INs and HBVs, followed by His299, His305, Asp300, and Arg195. As shown in Figure 3, these six amino acid molecules are in the largest cavity on the surface of the α-amylase molecule. This is usually the active site or substrate-binding site of the enzyme. Therefore, these residues are vital for α-amylase and inhibitory peptide binding and for the inhibitory activity of the peptides. Research has suggested that His201 plays a critical role during α-amylase inhibition. A study involving the α-amylase inhibitory mechanism via ferulic acid indicated that His201 and Ile235 surround the active α-amylase site [26], while research on hydrolyzed oat bran proteins and peptides showed that His201 belongs to the α-amylase calcium-binding domain [19]. Another study revealed that Tyr151, His201, and Ile235 represent the main bean-peptide-to-α-amylase binding sites during molecular docking [27]. These findings suggest that His201 may be critical for α-amylase inhibition. Furthermore, Asp300 is one of the three amino acid residues in the catalytic triad, which plays a vital role in α-amylase catalytic activity.

The types of amino acid residues on α-amylase were further analyzed; residue types are considered independent variables, while different amino acid residues of the same type are deemed sample points of independent repeated trials. The statistical results are shown in Table 4. The results for both INs and HBVs showed that amino acids with positively charged side chains had the highest total number of residue types, followed by polar uncharged amino acids, with positively charged amino acids significantly higher in HBVs than nonpolar amino acids (*p* < 0.05). This indicated that amino acids with positively charged side chains played a more important role during α-amylase and inhibitory peptide interactions.

### 2.3. Analysis of the Key Sites on the α-Amylase Inhibitory Peptides

To investigate the differences in the ability of different types of amino acids to form hydrogen bonds with α-amylase, the HBVs of each hydrogen-bond-forming amino acid were calculated, as presented in Table 5. The results showed that Asp had the highest HBVs, followed by Arg, both of which were significantly higher than Leu (*p* < 0.05). This implies that the Asp and Arg on the peptides may play a more prominent α-amylase inhibitory role. Studies have treated barley α-amylase/subtilisin inhibitors with reagents specific to several amino acids, showing that arginine is necessary for their inhibitory effect, consequently corroborating its importance in inhibitory peptides [28].

These amino acid residues can be categorized into four groups based on the polarity and charge of the side chains, allowing for the calculation of the mean HBV value of each amino acid type. The data in Table 5 are further summarized and presented in Table 6. Amino acids with positively and negatively charged side chains had significantly higher HBVs than nonpolar and uncharged amino acids (*p* < 0.05, Table 6). Thus, the charge of the peptide may affect the formation of hydrogen bonds between the α-amylase and the peptide, which is closely related to the inhibitory activity.

The amino acid residues on the α-amylase molecule with the highest HBV values are positively charged, whereas the inhibitory peptides with the highest HBV values are negatively charged, and the two will be attracted to each other, thereby increasing the possibility of hydrogen bond binding and inhibitory activity.

### 2.4. Analysis of Hydrogen Bond Distribution Characteristics on α-Amylase Inhibitory Peptides

On a peptide chain, amino acid residues closer to the N-terminal or C-terminal of the peptide chain may present different physicochemical properties. Similarly, the same is true for those closer to the terminal or center of a peptide chain. Research on flavor peptides suggests that the properties of some peptides are enhanced when specific amino acids are located at both ends of the sequences [29,30], while another study involving a predictive model for antioxidative peptides revealed that amino acid residues located near rather than at the C-terminal contribute more significantly to their activity [31]. Therefore, the physicochemical property variation caused by positional differences may affect the inhibitory effect of α-amylase inhibitory peptides. Since hydrogen bonds are associated with inhibitory activity, their distribution on the peptides was examined to determine which parts of the amino acid residues on α-amylase inhibitory peptides presented a more restrictive effect.

The analysis was performed according to the data presented in Table S3. One-sample *t*-tests were performed using the data shown in Table 7, with a test value of 0.5. The results showed that the *F* value, 0.747, was significantly higher than the test value, 0.5 (*p* < 0.05). This indicates that the hydrogen bond distribution on the α-amylase inhibitory peptides was significantly closer to the ends than the center. Therefore, the amino acid residues closer to the ends may be more important for determining the α-amylase inhibitory mechanism of the peptides than those near the center.

### 2.5. Molecular Dynamics Result Analysis of the α-Amylase Molecule and Its Inhibitory Peptides

To determine the effect of key binding sites on α-amylase stability, three peptides (KLPGF, PLPLH, and GNPVGGVGHGTTGT) that all included important residues (top 50% in both INs and HBVs) in the binding site were selected. The complexes formed by the three peptides with α-amylase were subjected to molecular dynamics simulations.

**Figure 4:**
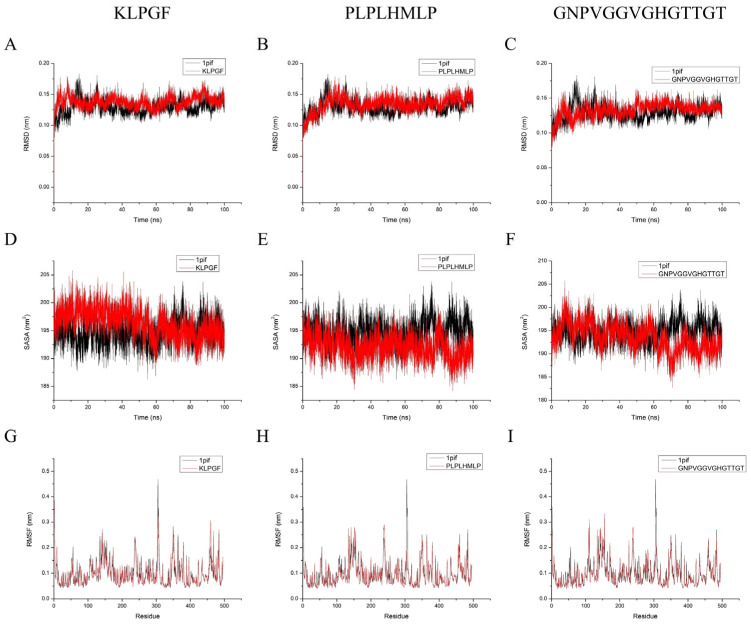
Molecular dynamics simulation (100 ns) outcomes for KLPGF, PLPLHMLP, and GNPVGGVGHGTTGT with α-amylase. (**A**–**C**) RMSD; (**D**–**E**) SASA; (**G**–**I**) RMSF. 1pif stands for α-amylase.

Three indicators, root mean square deviation (RMSD), solvent accessible surface area (SASA), and root mean square fluctuation (RMSF), were chosen to analyze the stability of these complexes. As shown in Figure 4A–C, the RMSD values of the four systems (α-amylase, three peptide-enzyme complexes) almost reached equilibrium at 100 ns of the simulation, indicating that all the simulated systems reached a steady state, so the kinetic simulation results are reliable and can be used for subsequent kinetic simulation analysis [32]. The RMSD values of all three peptide–enzyme complexes can reach a steady state faster and with less fluctuation than α-amylase alone, indicating that the binding of peptides to important residues of α-amylase can make α-amylase reach equilibrium more readily and have better stability [33]. At 60–100 ns, the SASA of the peptide–amylase complexes was progressively smaller than that of α-amylase alone (Figure 4D–F). This suggests that peptide binding to α-amylase leads to an increase in the hydrophobicity of its overall structure and a more stable structure in the solvent [34]. Furthermore, the fluctuations of α-amylase residues during the simulation were also analyzed. As shown in Figure 4G–I, the RMSF fluctuations of the important residues involved in the three peptide binding sites are less than those of α-amylase alone. This demonstrated that binding made the α-amylase critical residues less flexible and that they possessed better stability [35]. All the results indicate that the key site of α-amylase plays a critical role in allowing the peptide to exert its inhibitory activity.

The 80–100 ns segment was extracted for the binding free energy calculation based on prior molecular dynamics simulation trajectories, and the various energy terms are shown in Table 8. Negative values indicated that these energy terms promote the binding of α-amylase and peptides, with larger absolute values indicating stronger promoting effects. From a numerical perspective, the van der Waals energy (Δ*G*_vdw_) and the electrostatic energy (Δ*G*_ele_) are the main factors affecting the binding of peptides and proteins, with the nonpolar solvation energy (Δ*G*_nonpolar_) having a minor promoting influence. Conversely, the binding of proteins and peptides is negatively impacted by the polar solvation energy (Δ*G*_polar_) [36]. The total binding free energies (Δ*G*_total_) of KLPGF, PLPLHMLP, and GNPVGGVGHGTTGT to α-amylase were −50.26, −24.34, and −27.58 kcal/mol, respectively. The results of the binding free energy calculations showed that peptides can spontaneously bind to α-amylase and contribute favorably to α-amylase stability, and the role of key amino acids cannot be underestimated [37].

### 2.6. Analysis of the PLSR Results for the Physicochemical Properties and Inhibitory Peptide Activity

**Figure 5:**
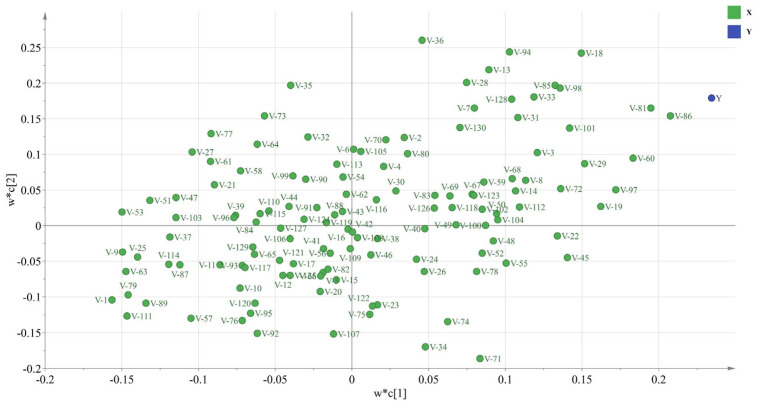
PLSR loading analysis diagram of physicochemical properties and inhibitory peptide activity. The numbers in the independent variable names were obtained by ordering the residue positions of the decapeptide and the serial numbers of the 13 descriptors. For example, V-13 represents the independent variable of the 13th descriptor of the first residue, and V-14 represents the independent variable of the first descriptor of the second residue.

Two principal components were selected to generalize the independent variables. The regression results yielded a coefficient of determination (*R^2^*) of 0.9044, indicating a good regression effect. The load analysis of the regression output from SIMCA-P is shown in Figure 5. The VIP values of 130 independent variables were obtained via regression calculation, among which, 52 independent variables were larger than one and were labeled significant independent variables. The V-86 and V-81 independent variables with the highest VIP values were V-86, V-81, etc., with VIP values of 2.053, 1.950, etc., respectively.

As shown in Table 9, the number of significant independent variables (NSIV) at the N-terminal was greater than at the C-terminal, indicating that the physicochemical properties of amino acids close to the N-terminal contributed more to the α-amylase inhibitory activity of the peptides than those near the C-terminal. Therefore, the amino acid residues in the third and fifth positions displayed a higher correlation with inhibitory activity, while those in the first position exhibited a lower association. Amino acid residues were further analyzed for hydrophobic, chargeability, hydrogen bond contributory, and steric properties. The results are shown in Table 10; the hydrogen bond contributory properties yielded the highest NSIV mean value. Consequently, it can be concluded that hydrogen bonds play the most important role among the several factors affecting α-amylase inhibitory peptide activity.

## 3. Materials and Methods

### 3.1. Materials

An α-amylase inhibitory peptide database was established using peptide sequences from the existing literature with pure product half-inhibitory concentrations (IC_50_) determined via in vitro experiments. For each peptide, the IC_50_ peptide concentration (mmol/L) was multiplied by the volume of the peptide solution (L) and then divided by the activity unit (U) of the α-amylase used in the experiment. Next, the uniform IC_50_ values were obtained in millimoles per activity unit (mmol/U), and their negative logarithms were calculated.

### 3.2. Molecular Docking of Food-Derived α-Amylase Inhibitory Peptides

Then, 3D information files for the peptides were generated online via the PEP-FOLD3 website (https://bioserv.rpbs.univ-paris-diderot.fr/services/PEP-FOLD3/, accessed on 11 October 2023). For every collected peptide, the structure with the lowest potential energy among several structures was selected [38]. The α-amylase crystal structure information file was obtained from the RCSB Protein Data Bank (http://www.rcsb.org/, accessed on 11 October 2023) with a pdb ID of 1pif [39].

AutoDock Vina 1.2.2 in the AutoDock Tools 1.5.6 software was used for docking [40]. The α-amylase molecules were removed from the water molecules, after which, charge and hydrogen atoms were added. The movable bonds of the peptides were set to the default mode; that is, the amide bonds were not rotatable, while the carbon backbone bonds were rotatable. The Center Grid Box formed the center of the macromolecule: center x = 33.29, center y = 26.47, and center z = 44.15. The center coordinates of the docking box are (33.29, 26.47, 44.15) for the axes (x, y, z). The docking box size was set to size x = 92 Å, size y = 126 Å, and size z = 106 Å, while the spacing was set to 0.55. The exhaustiveness parameter was 8. The result with the lowest binding energy was selected from several possible results obtained via the docking operation for each peptide. The docking results obtained via AutoDock Vina 1.2.2 were viewed using the PyMOL 2.5.5 software.

### 3.3. Docking Site Interaction Frequency of the α-Amylase Molecules

The interaction frequency of each residue on the α-amylase molecules was counted and summarized according to the type of amino acid side chain. When an inhibitory peptide interacts with a residue on an α-amylase molecule, one IN is recorded for the residue. A higher IN signifies an increased interaction frequency between the inhibitory peptide and the residue.

### 3.4. Hydrogen Bonding Qualities of the Docking Sites on the α-Amylase Molecules and Inhibitory Peptides

For the α-amylase molecules and inhibitory peptide molecules, the number of hydrogen bonds formed by each residue and the distances between them were analyzed, and HBVs were calculated according to Formula (1).

(1) $$HBV=\sum_{i=1}^{t}\frac{D}{d_{i}}$$

where *t* denotes the number of hydrogen bonds formed on the residue; *D* is the maximum bond length value of the formed hydrogen bonds, which is equal to 3.5 Å; and *d_i_* denotes the length of the *i*-th hydrogen bond.

### 3.5. Examination of the Hydrogen Bond Distribution Characteristics of the α-Amylase Inhibitory Peptides

The residues of each hydrogen bond on the peptide were recorded and listed with the position of the residue forming the hydrogen bond at the N-terminus of the peptide. The degree of hydrogen bonding close to the N- or C-terminals and the ends or center of the peptide were calculated, weighted with the hydrogen bond lengths. *R_N_* indicates the degree of hydrogen bonding close to the N-terminus of a specific peptide, and Formula (2) was used for the calculation.

(2) $$R_{N}=\frac{\sum_{i=1}^{m}\left(\frac{n_{i}\;-\;1}{L\;-\;1}\times d_{i}\right)}{\sum_{i=1}^{m}d_{i}}$$

where *n_i_* is the N-terminal position of the *i*-th hydrogen bond on a residue, *L* is the peptide length, *m* denotes the number of hydrogen bonds on the peptide, and di signifies the *i*-th hydrogen bond length. *R_N_* represents the N-terminal position value. An *R_N_* below 0.5 shows that the hydrogen bonds on the peptide are closer to the N-terminal, while a lower *R_N_* indicates the degree of closeness to the N-terminal. The distance between the hydrogen bonds on the inhibitory peptides and the N-terminal was determined after analyzing the significance between the *R_N_* value and 0.5.

Imitating Formula (2), the distance between the hydrogen bonds and the center of the peptide was determined and calculated as shown in Formula (3).

(3) $$F=\left\{\begin{array}{c} \frac{\sum_{i=1}^{m}\left[\left(1\;-\;\frac{\min\left(n_{i},c_{i}\right)\;-\;1}{\frac{L\;-\;1}{2}}\right)\;\times \;d_{i}\right]}{\sum_{i=1}^{m}d_{i}},\;while\;L\;is\;odd\\ \frac{\sum_{i=1}^{m}\left[\left(1\;-\;\frac{\min\left(n_{i},c_{i}\right)\;-\;1}{\frac{L\;-\;2}{2}}\right)\;\times \;d_{i}\right]}{\sum_{i=1}^{m}d_{i}},\;while\;L\;is\;even \end{array}\right.$$

where min (*n_i_*, *c_i_*) denotes the minimum value of the N- and C-terminal positions of the *i*-th hydrogen bond, and *F* is the near-median value. A lower near-median value indicated a higher degree of closeness to the center of the peptide. The distance between the hydrogen bonds on the inhibitory peptides and the center was determined after analyzing the significance between the *F* and 0.5 values.

### 3.6. Molecular Dynamics Simulation

PDB files for the inhibitory peptide–amylase complex and α-amylase generated by molecular docking were used as initial structures for molecular dynamics using Gromacs 2022.4. Amber14sb was selected for the force field, TIP3P was selected for the water molecule model, and each initial structure was placed in a square box. Water molecules and Na^+^ and Cl^−^ ions were used to solvate and neutralize the prepared system. After optimizing the system energy using the steepest descent minimization and conjugate gradient minimization, NVT and NPT simulations were performed at 200 ps, respectively. Finally, a formal simulation of 100 ns was performed. The simulation result files were analyzed using the analysis program in Gromacs 2022.4. The simulated trajectories were calculated for binding energy using the gmx_MMPBSA program (https://valdes-tresanco-ms.github.io/gmx_MMPBSA/dev/getting-started/, accessed on 21 December 2023) [41].

### 3.7. PLSR of the Physicochemical Peptide Properties and Inhibitory Activity

In total, 457 physicochemical parameters of 20 amino acids were collected from the AAindex database and divided into four types, hydrophobic, electrical, steric, and hydrogen bond contributory properties, for principal component analysis. Then, 13 parameters containing most of the valid information from the original data were selected and labeled amino acid descriptors [42]. Using two-terminal position numbering (TTPN) [43], five amino acid residues were extracted from both terminals of each peptide separately and spliced to form a decapeptide. Therefore, each sample contained 130 independent variables. A 20 × 130 matrix of independent variables was established, with each sample as a row and each independent variable as a column. Furthermore, a 20 × 1 dependent variable matrix was established with each sample as a row and the negative logarithm of the dependent-variable IC_50_ value as a column. The VIP values of the 130 independent variables were obtained after PLSR. Those with VIP > 1, considered more important in explaining the dependent variables [44], were labeled significant independent variables. The importance of the different inhibitory peptide amino acid positions was examined by counting the NSIV of each decapeptide amino acid residue. Similarly, the importance of different amino acid properties was assessed by counting the NSIV values of the 13 descriptors.

### 3.8. Statistical Analysis

Kruskal–Wallis and one-sample *t*-tests were employed for data analysis using the IBM SPSS Statistics 25 software, with *p* < 0.05 indicating significant differences. The graphs were prepared using Origin Pro (64-bit) SR2. The VIP value of each independent variable was calculated using the SICMA-P 11.5.0.0 software.

## 4. Conclusions

This study found that the amino acid residues involved in the binding of α-amylase to inhibitory peptides showed a well-defined pattern. Molecular docking simulated the binding of α-amylase to twenty inhibitory peptides, with a statistical investigation of related inhibitory activity. His201 and Gln63 in α-amylase and Asp and Arg in the peptides were found to be key sites for inhibition, with charged side chains being essential for activity restriction. Further molecular dynamics simulations of the complex stability showed that peptide binding to key sites considerably stabilized α-amylase, improving inhibition. PLSR analysis indicated that amino acid residues near the N-terminus and in the third and fifth positions of the inhibitory peptide were vital for α-amylase inhibition. Meanwhile, hydrogen bonding was found to greatly contribute to this inhibitory activity. We designed the statistical methods in this study and succeeded in identifying several statistically significant patterns in peptides with α-amylase inhibitory properties. This provides some theoretical groundwork for screening and designing peptides with antidiabetic activity. Nonetheless, this study has some limitations, such as the small number and short length of the peptides. In addition, more in vivo experiments are needed to validate the hypoglycemic effects of α-amylase inhibitory peptides.

## Figures and Tables

**Table 1:** A summary of the α-amylase inhibitory peptides.

No.	Sequence of Peptide	Peptide Length	IC_50_ Value (mmol/U)	Source	References
1	EAGVD	5	110.00	Egg	[16]
2	KLPGF	5	120.00	Egg	[16]
3	PPHMLP	6	103.69	Pinto bean	[17]
4	PPHMGGP	7	88.13	Pinto bean	[17]
5	LPLPLPLR	8	446.54	Camel milk	[18]
6	PLPLHMLP	8	70.22	Pinto bean	[17]
7	PLPWGAGF	8	69.91	Pinto bean	[17]
8	RALPIDVL	8	24.27	Oat bran	[19]
9	NINAHSVVY	9	22.43	Oat bran	[19]
10	RLARAGLAQ	9	69.88	Millet grain	[20]
11	IPLPLPLPLP	10	364.70	Camel milk	[18]
12	LRSELAAWSR	10	527.80	Spirulina platensis	[21]
13	YFDEQNEQFR	10	12.50	Oat bran	[19]
14	YGNPVGGVGH	10	80.28	Millet grain	[20]
15	GQLGEHGGAGMG	12	53.00	Millet grain	[20]
16	EQGFLPGPEESGR	13	51.09	Millet grain	[20]
17	GNPVGGVGHGTTGT	14	50.01	Millet grain	[20]
18	GEHGGAGMGGGQFQPV	16	44.58	Millet grain	[20]
19	HAGPTWNPISIGISFM	16	387.74	Camel milk	[18]
20	LSSLEMGSLGALFVCM	16	44.58	Pinto bean	[17]

**Table 2:** The INs of the amino acid residues on α-amylase.

Residue	Residue Type	IN	Residue	Residue Type	IN	Residue	Residue Type	IN
His201	Positive	15	Asp402	Negative	3	Val50	Nonpolar	1
Gln63	Uncharged	15	Asn53	Uncharged	3	Val51	Nonpolar	1
Asp300	Negative	14	Ser289	Uncharged	3	Pro54	Nonpolar	1
His305	Positive	14	Arg252	Positive	3	Glu149	Negative	1
His299	Positive	12	Thr6	Uncharged	2	Gly164	Uncharged	1
Val163	Nonpolar	11	Ser105	Uncharged	2	Leu165	Nonpolar	1
Trp59	Nonpolar	10	Ala198	Nonpolar	2	Ser226	Uncharged	1
Asp197	Negative	10	His331	Positive	2	Arg227	Positive	1
Tyr151	Uncharged	10	Glu352	Negative	2	Ala241	Nonpolar	1
Glu233	Negative	9	Arg398	Positive	2	Thr264	Uncharged	1
Lys200	Positive	9	Phe406	Nonpolar	2	Ser270	Uncharged	1
Arg195	Positive	9	Arg421	Positive	2	Glu272	Negative	1
His101	Positive	8	Glu282	Negative	2	Asp290	Negative	1
Asp356	Negative	8	Gly9	Uncharged	2	Phe335	Nonpolar	1
Leu162	Nonpolar	7	Gly106	Uncharged	2	Gly403	Uncharged	1
Tyr62	Uncharged	6	Tyr2	Uncharged	2	Asn152	Uncharged	1
Ile235	Nonpolar	6	Glu240	Negative	2	Pca1	—	1
Ala307	Nonpolar	6	Gln161	Uncharged	2	Val354	Nonpolar	1
Ile148	Nonpolar	4	Trp280	Nonpolar	2	Ala107	Nonpolar	1
Gly306	Uncharged	4	Arg291	Positive	2	Ser310	Uncharged	1
Gly308	Uncharged	4	Gln5	Uncharged	1	Ser311	Uncharged	1
Trp58	Nonpolar	3	Ser8	Uncharged	1	Ala3	Nonpolar	1
Gly309	Uncharged	3	Thr11	Uncharged	1	Thr52	Uncharged	1

**Table 3:** The HBVs of the amino acid residues on α-amylase.

Residue	Residue Type	HBV	Residue	Residue Type	HBV
Gln63	Uncharged	11.50	Trp280	Nonpolar	2.12
His201	Positive	10.33	Gln161	Uncharged	2.06
Arg252	Positive	10.00	Glu233	Negative	2.06
Arg195	Positive	6.06	Glu240	Negative	2.03
His299	Positive	5.19	Asn53	Uncharged	2.00
Asp356	Negative	5.18	Tyr2	Uncharged	1.75
Gly308	Uncharged	4.72	Ala3	Nonpolar	1.67
Arg291	Positive	4.45	Gly106	Uncharged	1.59
Gly306	Uncharged	4.34	Ser311	Uncharged	1.59
His305	Positive	4.30	Gly9	Uncharged	1.52
Thr52	Uncharged	4.20	Ser310	Uncharged	1.40
Asp300	Negative	4.19	Ala107	Nonpolar	1.25
Tyr151	Uncharged	3.85	Pca1	—	1.06
Lys200	Positive	3.59	Val354	Nonpolar	1.06
Asp197	Negative	3.29	Asp402	Negative	1.06
Ser289	Uncharged	3.05	Ile148	Nonpolar	1.03
Trp59	Nonpolar	2.87	Asn152	Uncharged	1.03

**Table 4:** The statistics of the INs and HBVs of the different residue types on α-amylase.

Side Chain Type	IN			HBV		
	Quantity of Residues	Total IN	Mean Value	Quantity of Residues	Total IN	Mean Value
Nonpolar	19	62	3.26	6	10.00	1.67 ^b^
Polar uncharged	26	72	2.77	14	44.60	3.19 ^ab^
Positively charged	12	79	6.58	8	46.67	5.83 ^a^
Negatively charged	11	63	5.73	7	18.81	2.69 ^ab^

^a,b^ indicate that the data are significantly different (*p* < 0.05).

**Table 5:** The HBVs of the amino acid residues on the inhibitory peptides.

Amino Acid Kind	Residue Type	Serial Number of Residues	HBV	Number of Amino Acids	Total HBV	Mean Value
Alanine	Nonpolar	7-6	1.40	13	7.81	0.60 ^ab^
		19-2	3.42			
		20-11	2.99			
Aspartate	Negative	1-5	6.00	3	10.24	3.41 ^a^
		8-6	1.40			
		13-3	2.84			
Glutamate	Negative	1-1	1.59	10	10.55	1.06 ^ab^
		12-4	1.59			
		13-4	1.84			
		16-1	1.59			
		18-2	2.94			
		20-5	1.00			
Phenylalanine	Nonpolar	2-5	5.35	8	11.69	1.46 ^ab^
		7-8	6.34			
Glycine	Uncharged	14-6	1.52	37	12.55	0.34 ^ab^
		15-10	1.52			
		15-8	1.59			
		15-12	2.12			
		17-8	1.40			
		17-10	1.35			
		18-4	1.30			
		20-7	1.75			
Histidine	Positive	3-3	1.35	9	11.23	1.25 ^ab^
		4-3	1.46			
		6-5	2.70			
		14-10	2.86			
		15-6	1.52			
		17-9	1.35			
Isoleucine	Nonpolar	11-1	1.00	6	1.00	0.17 ^ab^
Lysine	Positive	2-1	1.09	1	1.09	1.09 ^ab^
Leucine	Nonpolar	6-2	1.46	26	6.00	0.23 ^b^
		6-7	1.30			
Methionine	Nonpolar	19-16	1.06	8	4.71	0.59 ^ab^
		20-16	3.65			
Asparagine	Uncharged	13-6	1.00	6	4.15	0.69 ^ab^
		14-3	3.15			
Proline	Nonpolar	2-3	1.59	28	18.06	0.65 ^ab^
		3-6	3.78			
		3-6	3.78			
		4-7	1.06			
		6-1	1.06			
		6-8	1.59			
		11-10	5.20			
Arginine	Positive	5-6	1.84	8	12.06	1.51 ^a^
		5-8	1.03			
		8-1	3.09			
		10-4	1.06			
		12-2	1.94			
		12-10	1.00			
		13-10	3.93			
Serine	Uncharged	12-9	1.35	9	6.80	0.76 ^ab^
		19-14	2.86			
		20-2	1.35			
		20-3	1.25			
Threonine	Uncharged	17-14	3.82	4	3.82	0.96 ^ab^
Valine	Nonpolar	17-4	1.30	10	1.30	0.13 ^ab^
Tryptophan	Nonpolar	12-8	1.94	3	3.01	1.00 ^ab^
		19-6	1.06			
Tyrosine	Uncharged	13-1	1.00	3	4.65	1.55 ^ab^
		14-1	3.65			

^a,b^ indicate that the data are significantly different (*p* < 0.05). Since the amino acids cysteine and glutamine do not form hydrogen bonds, they are not listed in the table. The serial numbers in column 3 were obtained from two parts: the serial number of the peptide to which the residue belongs and the position of that residue on the peptide. For example, 7-6 represents the sixth residue in the No. 7 peptide. Residues with an HBV of 0 were also considered when calculating the quantity of a certain kind of amino acid on the peptides and its mean HBV, but these residues are not listed in columns 3 or 4 of the table.

**Table 6:** Statistics of the HBVs of different residue types on the inhibitory peptides.

Side Chain Type	Quantity of Residues	Total HBV	Mean Value
Nonpolar	102	50.32	0.49 ^b^
Polar uncharged	59	31.97	0.54 ^b^
Positively charged	18	26.22	1.46 ^a^
Negatively charged	13	20.79	1.60 ^a^

^a,b^ indicate that the data are significantly different (*p* < 0.05).

**Table 7:** The *R_N_* and *F* values of the inhibitory peptides and their mean values.

Peptides	*R_N_*	*F*
EAGVD	0.814	1.000
KLPGF	0.741	0.867
PPHMLP	0.864	0.774
PPHMGGP	0.719	0.719
LPLPLPLR	0.898	0.761
PLPLHMLP	0.495	0.563
PLPWGAGF	0.957	0.901
RALPIDVL	0.141	0.869
RLARAGLAQ	0.375	0.250
IPLPLPLPLP	0.760	1.000
LRSELAAWSR	0.684	0.693
YFDEQNEQFR	0.540	0.666
YGNPVGGVGH	0.347	0.780
GQLGEHGGAGMG	0.815	0.628
EQGFLPGPEESGR	0.000	1.000
GNPVGGVGHGTTGT	0.735	0.592
GEHGGAGMGGGQFQPV	0.115	0.754
HAGPTWNPISIGISFM	0.572	0.722
LSSLEMGSLGALFVCM	0.583	0.662
Mean value	0.587	0.747 ^a^
Test value	0.5	0.5 ^b^

^a,b^ indicate that the data are significantly different (*p* < 0.05).

**Table 8:** The binding free energies of three inhibiting peptide–amylase complexes.

Energy Component (kcal/mol)	KLPGF	PLPLHMLP	GNPVGGVGHGTTGT
Δ*G*_vdw_	−41.24	−39.93	−60.12
Δ*G*_ele_	−375.03	−118.30	−78.05
Δ*G*_polar_	372.90	138.72	117.78
Δ*G*_nonpolar_	−6.90	−4.83	−7.19
Δ*G*_gas_	−416.27	−158.23	−138.17
Δ*G*_solv_	366.00	133.89	110.59
Δ*G*_total_	−50.26	−24.34	−27.58

Δ*G*_vdw_: van der Waals energy; Δ*G*_ele_: electrostatic energy; Δ*G*_polar_: polar solvation energy; Δ*G*_nonpolar_: nonpolar solvation energy; Δ*G*_gas_: total gas phase free energy, Δ*G*_gas_ = Δ*G*_vdw_ + Δ*G*_ele_; Δ*G*_solv_: total solvation free energy, Δ*G*_solv_ = Δ*G*_polar_ + Δ*G*_nonpolar_; Δ*G*_total_: total binding free energy, Δ*G*_total_ = Δ*G*_gas_ + Δ*G*_solv_.

**Table 9:** The NSIV of the different amino acid residues.

Position End	1	2	3	4	5	Sum
N	6	6	9	3	7	31
C	1	3	5	6	6	21
Sum	7	9	14	9	13	52

**Table 10:** The total and mean NSIV values of the different amino acid properties.

Properties	Hydrophobic Properties	Electrical Properties	Hydrogen Bond Contributory Properties	Steric Properties
Descriptor quantity	2	4	2	5
Total value	6	13	14	19
Mean value	3.00	3.25	7.00	3.80

## Data Availability

Data are contained within the article and Supplementary Materials.

## Supplementary Materials

The following supporting information can be downloaded at https://www.mdpi.com/article/10.3390/molecules29020395/s1, Figure S1. Molecular docking results for all twenty peptides with α-amylase, including binding poses (left panel), interactions (middle panel), and the formation of hydrogen bonds (right panel). Table S1. Molecular docking results for twenty inhibitory peptides and α-amylase. Table S2. Residues on the α-amylase molecule that interact with the peptides. Table S3 The sites and lengths of the hydrogen bonds between the peptides and α-amylase.
