# Supplementary material for: Statistical Characterization of Food-Derived α-Amylase Inhibitory Peptides: Computer Simulation and Partial Least Squares Regression Analysis
Source: Molecules. 2024 Jan 13;29(2):395. doi: 10.3390/molecules29020395 (PMC10819330; doi:10.3390/molecules29020395)
Supplement: Supplementary file 1 [file molecules-29-00395-s001.zip › molecules-2786895-supplementary.pdf]

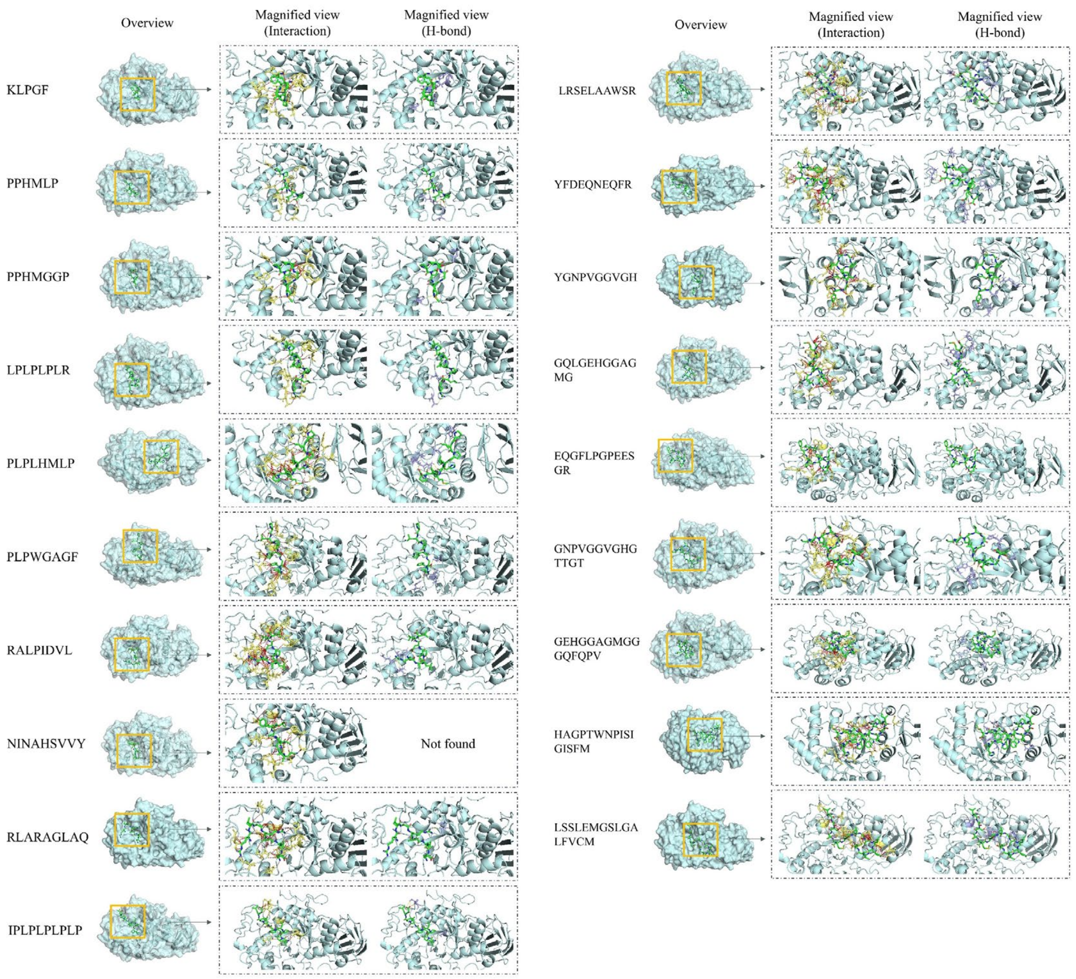

Figure S1. Molecular docking results for all twenty peptides with α-amylase include binding poses (left panel) as well as interactions (middle panel) and formation of hydrogen bonds (right panel). Blue molecules represent α-amylase, green molecules are peptide fragments, yellow molecules represent amino acids on α amylase that interact with peptide segments, red dashed lines indicate interactions, purple molecules represent amino acids on α amylase that form hydrogen bonds with the peptide segment, and yellow dashed lines indicate interactions.

Table S1. Molecular docking results of twenty inhibitory peptides and α-amylase.

Peptide	Mode	Affinity	Dist from best mode		Peptide	Mode	Affinity	Dist from best mode	
		kcal/mol	Rmsd l.b.	Rmsd u.b.			kcal/mol	Rmsd l.b.	Rmsd u.b.
EAGVD	1	-6.4	0.0	0.0	PLPLHMLP	1	-7.5	0.0	0.0
	2	-6.3	28.1	30.6		2	-7.5	2.9	6.1
	3	-6.3	27.7	30.8		3	-7.5	3.7	11.5
	4	-6.3	5.5	8.9		4	-7.4	31.3	34.1
	5	-6.2	27.9	30.7		5	-7.3	30.4	32.8
	6	-6.1	28.8	31.5		6	-7.2	2.0	4.2
	7	-6.1	4.4	9.0		7	-7.2	3.8	12.2
	8	-6.1	5.3	7.9		8	-7.2	30.9	33.6
	9	-6.0	28.6	30.7		9	-7.1	1.6	2.2
KLPGF	1	-6.9	0.0	0.0	PLPWGAGF	1	-8.7	0.0	0.0
	2	-6.5	3.5	7.2		2	-8.5	3.3	11.7
	3	-6.4	2.7	5.8		3	-8.2	3.1	12.6
	4	-6.4	2.4	5.2		4	-8.0	5.5	8.5
	5	-6.4	2.3	6.5		5	-7.6	32.1	37.3
	6	-6.3	27.0	29.5		6	-7.6	5.1	9.6
	7	-6.2	2.4	6.1		7	-7.5	31.9	37.0
	8	-6.1	27.9	29.6		8	-7.4	31.4	36.7
	9	-6.0	4.9	8.5		9	-7.4	3.3	11.6
PPHMLP	1	-7.5	0.0	0.0	RALPIDVL	1	-7.3	0.0	0.0
	2	-7.0	27.3	29.9		2	-7.3	6.1	11.8
	3	-6.8	27.3	30.0		3	-7.0	3.8	7.3
	4	-6.7	28.2	30.2		4	-7.0	29.9	32.0
	5	-6.7	7.3	12.2		5	-6.9	30.0	32.5
	6	-6.6	27.5	29.9		6	-6.8	29.6	32.2
	7	-6.5	28.0	29.6		7	-6.8	4.2	11.2
	8	-6.5	3.7	5.8		8	-6.5	3.8	7.7
	9	-6.5	3.6	7.0		9	-6.5	29.9	33.0
PPHMGGP	1	-7.8	0.0	0.0	NINAHSVVY	1	-7.4	0.0	0.0
	2	-7.4	28.6	30.8		2	-7.4	1.3	2.2
	3	-7.2	5.6	9.9		3	-7.4	29.5	33.4
	4	-7.0	4.5	9.8		4	-7.1	1.8	3.0
	5	-6.9	2.9	9.1		5	-7.1	29.6	33.8
	6	-6.9	29.3	31.3		6	-7.0	2.6	3.9
	7	-6.9	28.8	31.6		7	-6.9	33.3	37.0
	8	-6.8	29.0	31.4		8	-6.9	4.4	10.6
	9	-6.8	28.9	31.6		9	-6.8	29.3	32.5
LPLPLPLR	1	-7.5	0.0	0.0	RLARAGLAQ	1	-6.5	0.0	0.0
	2	-7.4	1.8	3.7		2	-6.5	2.8	6.7
	3	-7.3	5.9	13.6		3	-6.5	3.1	5.8

	4	-7.2	27.3	31.7		4	-6.4	2.7	12.9
	5	-7.2	2.3	14.2		5	-6.4	5.3	9.2
	6	-7.2	27.2	31.1		6	-6.3	5.3	9.5
	7	-7.1	3.7	8.3		7	-6.2	2.5	12.3
	8	-7.1	27.0	31.3		8	-6.2	34.2	38.1
	9	-7.0	27.4	31.5		9	-6.2	34.6	37.8
	1	-7.8	0.0	0.0		1	-8.5	0.0	0.0
	2	-7.6	35.0	39.2		2	-8.3	2.5	3.9
	3	-7.6	4.1	7.6		3	-8.1	31.6	37.1
IPLPLPLP LP	4	-7.6	6.0	11.7	EQGFL PGPEES GR	4	-7.7	32.3	37.7
	5	-7.6	6.1	11.3		5	-7.7	32.3	38.1
	6	-7.5	8.7	18.3		6	-7.6	3.9	9.6
	7	-7.4	7.1	17.3		7	-7.2	29.7	35.8
	8	-7.4	4.2	8.7		8	-7.2	8.7	15.5
	9	-7.4	35.2	39.4		9	-7.1	3.3	6.7
	1	-6.7	0.0	0.0		1	-7.8	0.0	0.0
	2	-6.5	30.4	33.6		2	-7.7	3.9	7.4
	3	-6.5	29.9	32.8		3	-7.7	29.8	34.9
LRSELAA WSR	4	-6.4	30.0	33.7	GNPVG GVGH GTTGT	4	-7.6	30.7	33.9
	5	-6.3	29.8	33.8		5	-7.6	30.4	35.4
	6	-6.3	30.5	33.5		6	-7.5	3.5	6.5
	7	-6.3	29.8	34.6		7	-7.5	30.6	34.8
	8	-6.3	30.1	33.8		8	-7.3	5.4	12.3
	9	-6.2	3.0	7.5		9	-7.3	3.1	8.7
	1	-7.1	0.0	0.0		1	-7.3	0.0	0.0
	2	-6.9	1.9	5.0		2	-7.3	31.0	36.2
	3	-6.8	3.5	8.4		3	-7.2	31.2	37.3
YFDEQNE QFR	4	-6.8	6.0	11.7	GEHGG AGMG GGQFQ PV	4	-7.1	35.0	42.1
	5	-6.7	32.6	36.9		5	-7.0	32.2	36.9
	6	-6.7	6.1	11.6		6	-7.0	30.9	35.0
	7	-6.6	33.6	37.7		7	-7.0	39.3	45.3
	8	-6.5	5.6	14.2		8	-7.0	30.6	35.0
	9	-6.5	2.6	6.6		9	-6.9	30.9	35.3
	1	-7.0	0.0	0.0		1	-7.0	0.0	0.0
	2	-6.9	9.9	15.3		2	-6.8	3.8	9.0
	3	-6.8	29.8	34.3		3	-6.8	3.8	14.0
YGNPVGG VGH	4	-6.8	32.9	37.4	HAGPT WNPISI GISFM	4	-6.8	33.0	37.3
	5	-6.6	30.8	35.0		5	-6.8	28.2	32.6
	6	-6.6	30.8	35.0		6	-6.8	1.7	2.3
	7	-6.6	10.0	15.5		7	-6.7	32.1	37.1
	8	-6.4	31.6	36.0		8	-6.5	3.4	14.0
	9	-6.4	29.1	32.5		9	-6.5	1.9	3.0
GQLGEHG GAGMG	1	-7.0	0.0	0.0		1	-7.1	0.0	0.0
	2	-7.0	35.0	39.2		2	-6.8	1.8	2.1

	3	-7.0	31.4	34.4		3	-6.8	1.7	2.1
	4	-7.0	31.4	34.8		4	-6.4	29.6	36.6
	5	-6.9	35.0	39.1	LSSLEM	5	-6.2	27.1	30.4
	6	-6.9	34.2	38.2	GSLGA	6	-6.2	28.0	32.9
	7	-6.8	1.3	2.3	LFVCM	7	-6.1	26.8	31.7
	8	-6.8	31.3	34.8		8	-6.1	29.4	36.6
	9	-6.7	31.1	34.2		9	-6.1	27.9	32.0

Table S2. Residues on the α-amylase molecule that interact with the peptides.

Peptide	The sites on the enzyme molecule where the interactions occur
EAGVD	Trp58, Tyr62, Gln63, Arg195, Asp197, His201, His299, Asp300, His305
KLPGF	Tyr62, His101, Leu162, Val163, Arg195, Asp197, Ala198, Lys200, His201, Glu233, Ile235, His299, Asp300, His305
PPHMLP	Gln63, Tyr151, Leu162, Arg195, Lys200, His201, Glu233, Glu240, His299, Gly308
PPHMGGP	Gln63, His101, Leu162, Arg195, His201, Glu233, His299, His305, Asp356
LPLPLPLR	Tyr62, Gln63, Tyr151, Asn152, Leu162, Lys200, His201, Glu233, Ile235, Glu240, Ala241, His299, Asp300, His305, Gly306
PLPLHMLP	Tyr2, Thr6, Gly9, Arg252, Trp280, Glu282, Ser289, Arg291, His331, Pro332, Phe335, Arg398, Asp402, Phe406, Arg421
PLPWGAGF	Trp59, Gln63, Tyr151, Val163, Arg195, Asp197, Ala198, His201, Glu233, His299, Asp300, His305, Glu352, Trp357
RALPIDVL	Trp58, Trp59, Gln63, His101, Ile148, Tyr151, Gln161, Leu162, Val163, Gly164, Leu165, Lys200, His201, Glu233, Asp300, His305, Asp356
NINAHSVVY	Trp59, Gln63, His101, Tyr151, Val163, Asp197, Lys200, His201, Glu233, Ile235, Asp300, His305, Ala307
RLARAGLAQ	Asn53, Trp59, Gln63, Ser105, Gly106, Gln161, Val163, Arg195, Asp197, His201, His299, Asp300, Asp356
IPLPLPLPLP	Val50, Val51, Thr52, Asn53, Trp59, Gln63, His101, Asp197, Asp300, His305
LRSELAAWSR	Trp58, Gln63, His101, Tyr151, Val163, Asp197, Lys200, His201, Ile235, His299, Asp300, His305, Ala307, Gly308, Gly309, Asp356
YFDEQNEQFR	Asn53, Trp59, Tyr62, Gln63, Ser105, Gly106, Ala107, Tyr151, Leu162, Val163, Lys200, His201, His299, Asp300, His305, Asp356
YGNPVGGVGH	Thr6, Ser8, Gly9, Arg252, Trp280, Glu282, Ser289, His331, Pro332, Gly334, Arg398, Asp402, Gly403, Arg421
GQLGEHGGAGMG	Trp59, Tyr62, Gln63, Ile148, Val163, Arg195, Asp197, His201, Glu233, Ile235, Asp300, His305, Ala307, Glu352, Val354, Asp356
EQGFLPGPEESGR	Trp59, Gln63, Ile148, Glu149, Tyr151, Val163, Lys200, His201, Ile235, Gly306, Ala307
GNPVGGVGHGTTGT	Trp59, Tyr62, Gln63, His101, Tyr151, Leu162, Val163, Arg195, Asp197, His201, His299, Asp300, His305, Gly306, Gly308, Gly309, Asp356
GEHGGAGMGGGQFQPV	Gln63, His101, Ile148, Tyr151, Val163, Arg195, Asp197, Lys200, His201, Glu233, His299, Asp300, His305, Ala307
HAGPTWNPISIGISFM	Pca1, Tyr2, Ala3, Gln5, Thr11, Ser226, Arg227, Arg252, Ser289, Asp290, Arg291, Pro332, Asp402, Phe406
LSSLEMGSLGALFVCM	Pro54, Trp59, Thr264, Ser270, Glu272, His299, Asp300, His305, Gly306, Ala307, Gly308, Gly309, Ser310, Ser311, Asp356

Table S3. The sites and lengths of the hydrogen bonds between the peptides and α-amylase.

Peptide	Peptide length	Site on enzyme molecule	Site on peptide molecule	Length of hydrogen bond (Å)
EAGVD	5	Gln63	E1	2.2
		Arg195	D5	2.2
		Asp197	D5	3
		His299	D5	1.9
		His299	D5	2.5
KLPGF	5	Arg195	F5	2.6
		Asp197	F5	3.4
		His201	P3	2.2
		Glu233	F5	3.4
		His299	F5	1.8
		Asp300	K1	3.2
PPHMLP	6	Lys200	P6	2
		His201	H3	2.6
		Glu240	P6	3.4
		Glu240	P6	3.5
PPHMGGP	7	His201	H3	2.4
		Asp356	P7	3.3
LPLPLPLR	8	Asn152	R8	3.4
		Lys200	R6	1.9
PLPLHMLP	8	Arg252	H5	2.5
		Arg252	H5	2.7
		Arg252	L7	2.7
		Trp280	P1	3.3
		Ser289	L2	2.4
		Arg291	P8	2.2
PLPWGAGF	8	Arg195	F8	2.1
		Arg195	F8	2.4
		Asp197	F8	3.2
		His201	A6	2.5
		Glu233	F8	3.4
		Asp300	F8	3.2
RALPIDVL	8	His101	D6	2.5
		Ile148	R1	3.4
		Gln161	R1	3.4
		Gln161	R1	3.4
NINAHSVVY	9	—	—	—
RLARAGLAQ	9	Asp356	R4	3.3
IPLPLPLPLP	10	Thr52	P10	2.2
		Thr52	P10	2.5
		Thr52	P10	2.9
		Asn53	P10	3.5
		Asp300	I1	3.5
LRSELAAWSR	10	Gln63	W8	1.8
		Gln63	S9	2.6
		His305	R2	1.8
		Gly308	E4	2.2
		Asp356	R10	3.5
YFDEQNEQFR	10	Asn53	Y1	3.5
		Gln63	E4	1.9
		Gly106	D3	2.2
		Ala107	D3	2.8
		Tyr151	R10	2.9
		His201	R10	2.1

YGNPVGGVGH	10	His305	R10	3.3
		Asp356	N6	3.5
		Gly9	G6	2.3
		Arg252	N3	2
		Arg252	N3	2.5
		Arg252	H10	2.4
		Arg252	H10	2.5
		Trp280	Y1	3.3
		Glu282	Y1	3.5
		Ser289	Y1	2.2
GQLGEHGGAGMG	12	Trp59	G10	2.3
		Gln63	G8	2.2
		His201	H6	2.3
		Val354	G12	3.3
		Asp356	G12	3.3
EQGFLPGPEESGR	13	Gln63	E1	2.2
GNPVGGVGHGTTGT	14	His101	G8	2.5
		Tyr151	G10	2.6
		His201	H9	2.6
		His305	V4	2.7
		Gly306	T14	3.2
		Gly306	T14	3.3
		Gly308	T14	2.1
GEHGGAGMGGGQFQPV	16	Gln63	E2	2.2
		His101	E2	2.6
		Tyr151	G4	2.7
		Pca1	M16	3.3
HAGPTWNPISIGISFM	16	Tyr2	A2	2
		Ala3	A2	2.1
		Arg291	S14	2.4
		Arg291	S14	2.5
		Asp402	W6	3.3
LSSLEMGSLGALFVCM	16	Trp59	S2	2.6
		Asp300	E5	3.5
		Gly305	G7	2
		Gly305	S3	2.8
		Gly306	M16	3.1
		Gly306	M16	3.3
		Gly308	M16	2.4
		Ser310	A11	2.5
		Ser311	A11	2.2
